# SPOT: spatial proteomics through on-site tissue-protein-labeling

**DOI:** 10.1186/s12014-024-09505-5

**Published:** 2024-10-24

**Authors:** Yuanwei Xu, T. Mamie Lih, Angelo M. De Marzo, Qing Kay Li, Hui Zhang

**Affiliations:** 1 Department of Pathology, Johns Hopkins University School of Medicine, Baltimore, MD USA; 2 Department of Chemical and Biomolecular Engineering, Johns Hopkins University, Baltimore, MD USA; 3 Department of Oncology, Sidney Kimmel Cancer Center at Johns Hopkins Medical Institutions, Baltimore, MD USA; 4 Department of Urology, Johns Hopkins University School of Medicine, Baltimore, MD USA

**Keywords:** Spatial proteomics, Tissue-protein-labeling, Prostate cancer, Mass spectrometry

## Abstract

**Background:**

Spatial proteomics seeks to understand the spatial organization of proteins in tissues or at different subcellular localization in their native environment. However, capturing the spatial organization of proteins is challenging. Here, we present an innovative approach termed Spatial Proteomics through On-site Tissue-protein-labeling (SPOT), which combines the direct labeling of tissue proteins in situ on a slide and quantitative mass spectrometry for the profiling of spatially-resolved proteomics.

**Materials and Methods:**

Efficacy of direct TMT labeling was investigated using seven types of sagittal mouse brain slides, including frozen tissues without staining, formalin-fixed paraffin-embedded (FFPE) tissues without staining, deparaffinized FFPE tissues, deparaffinized and decrosslinked FFPE tissues, and tissues with hematoxylin & eosin (H&E) staining, hematoxylin (H) staining, eosin (E) staining. The ability of SPOT to profile proteomes at a spatial resolution was further evaluated on a horizontal mouse brain slide with direct TMT labeling at eight different mouse brain regions. Finally, SPOT was applied to human prostate cancer tissues as well as a tissue microarray (TMA), where TMT tags were meticulously applied to confined regions based on the pathological annotations. After on-site direct tissue-protein-labeling, tissues were scraped off the slides and subject to standard TMT-based quantitative proteomics analysis.

**Results:**

Tissue proteins on different types of mouse brain slides could be directly labeled with TMT tags. Moreover, the versatility of our direct-labeling approach extended to discerning specific mouse brain regions based on quantitative outcomes. The SPOT was further applied on both frozen tissues on slides and FFPE tissues on TMAs from prostate cancer tissues, where a distinct proteomic profile was observed among the regions with different Gleason scores.

**Conclusions:**

SPOT is a robust and versatile technique that allows comprehensive profiling of spatially-resolved proteomics across diverse types of tissue slides to advance our understanding of intricate molecular landscapes.

**Supplementary Information:**

The online version contains supplementary material available at 10.1186/s12014-024-09505-5.

## Background

The orchestra of different molecules within cells and cells within tissues is a crucial context for effective cellular and tissue functions. As the workhorses of the cells, proteins carry out diverse functions in different cellular or subcellular locations [1]. The exclusiveness of these protein locations allows for multiple cellular reactions to occur in parallel while avoiding undesirable cross-talk. Chemical environments also differ in these cellular or subcellular locations with corresponding interactors tailored to specific protein functions [2]. Certain proteins even shuttle in and out of different locations, they are called “shuttling proteins”, also known as multi-localized proteins [1, 2]. Mislocation and altered dynamics of proteins could compromise their proper cellular functions. The alterations in protein functions or locations could associated with diseases, such as metabolic disorders, neurodegenerative diseases, and cancers [3–6]. Notably, over 150 human disorders arise from disruptions in intracellular protein transport [7]. Spatial proteomics offers a way to capture the intricate spatial distribution of proteins, and potentially refresh our knowledge of cellular biology and disease pathogenesis, thereby guiding the development of new diagnostic and therapeutic strategies.

Proteomics studies, up until recently, have predominantly concentrated on profiling bulk tissues or dissociated cells. However, this approach inevitably results in a loss of the spatial context of proteins within cells, as well as of cells within tissues. Spatially resolved proteomic methods could address this challenge, enabling proteome-scale measurements while preserving spatial context. Conventionally, the study of protein localization and function has been confined to techniques such as immunofluorescence and electron microscopy, which provide limited information on the distribution and interaction of individual proteins within cells. However, recent advances in proteomic technologies, such as multiplexed imaging techniques and mass spectrometry (MS) [8, 9] have enabled the comprehensive profiling of the cellular distribution and regulation of proteins in complex biological systems.

Multiplex imaging techniques that leverage immunohistochemistry (IHC) [10], immunofluorescence (IF) [11–13], multiplexed ion beam imaging (MIBI) [14, 15], and imaging mass cytometry (IMC) [16, 17] have been developed for the determination of protein localization, visualization of protein organization, and interactions within tissues or cells. Currently, up to 65 antigens could be simultaneously detected using an iterative immunolabeling and chemical bleaching method [11]. Nevertheless, multiplex imaging techniques are highly reliant on the availability of suitable tagging antibodies, and the adoption of multiplexable signal amplification methods [18]. Alternatively, label-free mass spectrometry imaging (MSI) can directly detect the spatial distribution of molecules based on their mass-to-charge ratio (m/z) without the need for prior labeling with antibodies or other chemical tags [19, 20]. Mainstream techniques include matrix-assisted laser desorption/ionization (MALDI) [21–24], desorption electrospray ionization (DESI) [25], secondary ion mass spectrometry (SIMS) [26] and laser ablation electrospray ionization (LAESI) [27]. Despite the unlimited multiplexing capabilities of label-free MSI techniques, they often lack in specificity [28]. New imaging techniques such as MALDI-IHC [28, 29], which harness the specificity of antibodies or lectins conjugated with photocleavable mass-tags (PC-MTs), alongside the sensitivity and multiplexing capabilities of mass spectrometry, have revolutionized the field of spatial proteomics.

Imaging techniques for spatial proteomic studies are superior in resolution, specificity and sensitivity, yet they often face limitations in proteome coverage. In contrast, bottom-up MS-based spatial proteomics has evolved as a robust approach that involves the identification and quantification of proteins within different subcellular compartments or structures. Various methods have been coupled with MS for spatial proteomics, immunoprecipitation [30], proximity labeling [31, 32], and laser microdissection (LMD) [33, 34]. Immunoprecipitation isolates proteins that are associated with or inside organelles via selective antibody purification of target proteins that are fused with a tag. Proximity labeling is a more recently developed approach. It fuses proteins, which are in close proximity to the “bait protein(s)”, with an enzyme that can catalyze the covalent labeling of nearby proteins with a small molecule (e.g., biotin). The labeled proteins can then be isolated using avidin or streptavidin affinity purification. The proteins extracted from the immunoprecipitation and proximity labeling can be analyzed using MS to identify the interacting proteins and their subcellular localization. LMD coupled with MS is a widely adopted technique. LMD empowers researchers to meticulously dissect and capture specific cells or regions of interest within complex tissue samples while preserving their spatial context [35]. The isolated cells or regions subsequently undergo MS analysis to identify and quantify the proteins present within that precise spatial context [33, 34].

In general, imaging methods preserve the native environment but they are limited in depth, while current MS-based methods require spatially enriched samples out of their original context but excel in the deep profiling of the proteomics. Therefore, we have developed Spatial Proteomics through On-site Tissue-protein-labeling (SPOT) to achieve deep proteomic profiling of tissue proteins while retaining their spatial context. SPOT utilizes on-site labeling of the tissue proteins and MS for protein quantification and identification, respectively. Mouse brain tissue slides were first used to demonstrate that SPOT can identify proteins in different cellular compartments. Furthermore, we extended the application of SPOT to frozen tissues on slides and FFPE tissues on TMAs from prostate cancer, where a distinct proteomic profile was observed among the regions with different Gleason scores. The result indicates that the application of on-site labeling with TMT in spatial proteomics has the potential to reveal new insights into the subcellular localization and regulation of proteins in a wide range of biological processes, including development, disease, and cellular signaling.

## Methods

### Tissue sample collection and preparation

Mouse brain slides (7 μm) were purchased from Zyagen (San Diego, California. MF-201-HS for frozen slides and MP-201-SS for FFPE slides). Briefly, frozen mouse brain slides were air-dried to remove moisture and stained with 0.1% Mayer’s hematoxylin (Sigma, MHS32) for 10 min in a 50 mL conical tube. Then rinse in warm running tap water for 15 min for the “bluing” of the slides. Following this, slides were then dipped in ddH_2_O for 30 s. For eosin staining, air-dried non-stained or H-stained slides were placed in 95% reagent alcohol (Sigma, R8382) for 30 s, then transferred to eosin Y alcoholic solution (Sigma, HT1101) for 60 s. Stained slides were dehydrated through 2 changes each of 95% reagent alcohol, 100% reagent alcohol and xylene for 2 min each. No cover slips were mounted.

Fresh frozen prostate cancer tissue samples were obtained from JHU Pathology Core/Biospecimen Bank with approval from the Institutional Review Board of Johns Hopkins Medical Institutions. A standard tissue collection procedure was used. Briefly, prostate cancer tissue specimens were immediately embedded in optimal cutting temperature (OCT) compound and snap-frozen (as tissue block) in liquid nitrogen [36]. Frozen tissue blocks were stored at −80 °C until further processing. The Sects. (4–5 µm) were cut using a cryostat and mounted onto glass slides. Fresh frozen prostate cancer tissue slides were stained by hematoxylin and eosin (H&E) following aforementioned procedure for morphological evaluations [37]. The targeted areas were identified by a pathologist.

Prostate cancer tissue microarrays (TMA) were constructed using FFPE tissue blocks obtained from surgically resected prostate cancer. In the TMA, representative cancer areas were extracted as small cores (0.6 mm), and then embedded into a new TMA block. 5 μm sections were cut from the TMA block and used for the experiment [30]. To remove the paraffin from FFPE sagittal mouse brain slides, FFPE slides were first baked in an oven at 60 °C for 10 min, and soaked in xylene (10 min X 2). The slides were then subject to serial washes of 100% ethanol (5 min X 1), 70% ethanol (5 min X 1), 50% ethanol (5 min X 1) and HPLC-grade water (5 min X 1). To decrosslink proteins, deparaffinized slides were incubated in pH 8.0 100 mM Tris buffer at 70 °C for 20 min, washed with 1X PBS buffer (3 min X 1) and HPLC-grade water (3 min X 1), and dried with nitrogen gas in the end.

### Tissue sample annotation

Mouse brain atlas was determined using available data archived in Allen Brain Atlas [38] as well as MRI images generated previously [39]. For the prostate tissue slides, Gleason scores of the prostate cancer were re-reviewed by the American Board of Pathology certified pathologist, who has experience with prostate cancer. The targeted areas with different Gleason scores were selected and marked on the H&E slides. In addition, the slides were assessed under the light microscope at various magnifications, including low power (e.g., 4 × or 10x) for overall tissue architecture assessment and high power (e.g., 20 × or 40x) for detailed cytological characterization. The International Society of Urological Pathologycriteria and guidelines for the classification of prostate cancer were used (ISUP [40]). In our study, all Gleason scores of 3 to 5 were included.

### Tissue sample labeling using TMT

Each TMT reagent (Thermo Scientific) vial was carefully opened, and the contents were gently suspended using the recommended volume of anhydrous acetonitrile by the manufacturer. The reagent was mixed thoroughly to ensure complete dissolution.

Suspended TMT reagents were diluted 1:5 using 500 mM HEPES (TMT final concentration was 10 µg/µL in 100 mM HEPES) and applied directly to areas of interest using the pipette. The same procedure would be repeated for a total of 5 times, and between each pipetting, sections with labeling reagent would be left to air-dry. After labeling the sections of interest 5 times, 5% hydroxylamine was applied similarly to quench the labeling.

### Tissue lysis and digestion

Labeled tissue samples were scraped off the slides using a scalpel and subjected to 8 M urea lysis buffer (8 M urea, 75 mM NaCl, 50 mM Tris–HCl, pH8). Enzymatic tryptic digestion was performed as previously described [41]. Digested tissue samples were cleaned up by SCX tip, desalted by C18 StageTip, and dried using Speed-Vac.

### LC–MS/MS analysis

The analytical column was manufactured in-house using ReproSil-Pur 120 C18-AQ 1.9 μm stationary phase (Dr. Maisch GmbH) and slurry packed into a 28-cm length of 360 μm o.d. × 75 μm i.d. fused silica picofrit capillary tubing (New Objective). The analytical column was heated to 50 °C using a column heater (Phoenix-ST). The analytical column was equilibrated to 98% Mobile Phase A (MP A, 3% (v/v) ACN, 0.1% (v/v) FA) and 2% Mobile Phase B (MP B, 90% (v/v) ACN, 0.1% (v/v) FA) and maintained at a constant column flow of 200 nL/min. The sample was injected into a 12 μL loop placed in line with the analytical column which initiated the gradient profile (min:%MP B): 0:2, 1:6, 85:30, 94:60, 95:90, 100:90, 101:50, 110:50. The column was allowed to equilibrate at start conditions for 30 min between analytical runs.

MS analysis was performed using an Orbitrap Fusion Lumos mass spectrometer (Thermo Fisher Scientific). Electrospray voltage (1.8 kV) was applied at a carbon composite union (Valco Instruments) coupling a 360 μm o.d. × 20 μm i.d. fused silica extension from the LC gradient pump to the analytical column and the ion transfer tube was set at 250 °C. Following a 25 min delay from the time of sample injection, Orbitrap precursor spectra (AGC 4E5) were collected from 350–1800 m/z for 110 min at a resolution of 60 K along with data-dependent Orbitrap HCD MS/MS spectra (centroid) at a resolution of 50 K (AGC 1E5) and max injection time of 105 ms for a total duty cycle of 2 s. Masses selected for MS/MS were isolated (quadrupole) at a width of 0.7 m/z and fragmented using a collision energy of 37%. Peptide mode was selected for monoisotopic precursor scan and charge state screening was enabled to reject unassigned 1 + , 7 + , 8 + , and > 8 + ions with a dynamic exclusion time of 45 s to discriminate against previously analyzed ions between ± 10 ppm.

### Database search and data analysis

All raw files were processed through MS-PyCloud [42] that were converted into mzML and searched against Mus musculus (for mouse brain data) and Homo sapiens (for prostate cancer data) protein sequences downloaded from UniProt/Swiss-Prot via MS-GF + using the following settings: fixed modification of carbamidomethyl at cysteine, dynamic modifications of oxidation at methionine and TMT at lysine and protein N-terminus, precursor mass tolerance of 20 ppm, miss cleavages ≤ 2, instrument ID of “High-res LTQ,” and fragmentation method of HCD. A false discovery rate of 1% at the PSM level, a minimum of 1 PSM per peptide, and a minimum of 1 peptide per protein were required. MS-PyCloud search results can be found in Table S1-S3 in the supplementary.

Protein abundances were calculated by summing up the abundances of peptides belonging to the same protein. Median normalization was carried out for each TMT channel. Proteins with over 50% missing values were omitted. For differential analysis, median-normalized datasets were further log2 transformed. Pair-wise comparisons were conducted using the Wilcoxon ranked sum test, and proteins with a p-value less than 0.05 and an absolute fold-change greater than 2 were considered significantly differentially expressed.

Identified differentially expressed proteins from the prostate datasets were matched to the normal prostate proteome (126 genes enriched in prostate, Human Proteome Atlas [2, 43]), prostate cancer proteome (134 genes related to poor prostate cancer prognosis, Human Proteome Atlas [2, 43]), and cell markers in the prostate (199 genes, Cell Marker 2.0) [44].

## Results

### Design of SPOT

**Fig. 1:**
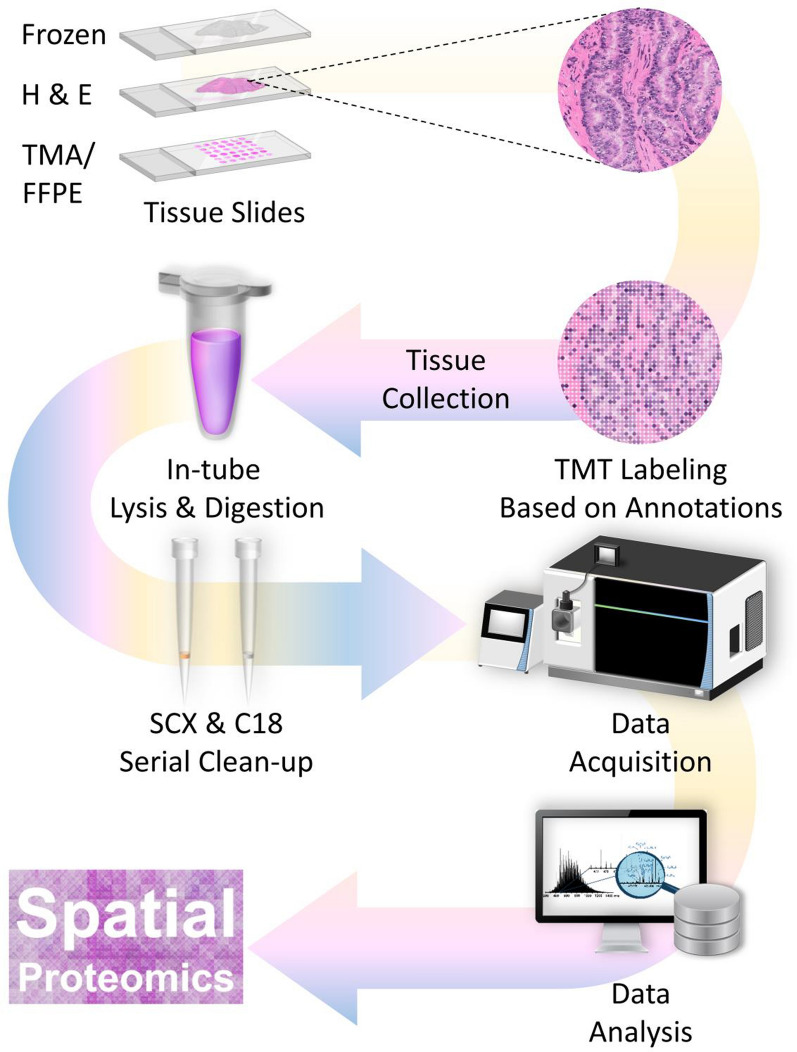
Overview of the SPOT workflow. Tissue slides are first annotated by cell types, histological patterns, or pathological states, followed by applying TMT tags directly onto regions of interest. After on-slide labeling and quenching of TMT, the tissue would be lysed, digested, and cleaned up for the downstream proteomic analysis using a mass spectrometer

SPOT is designed to provide quantitative deep profiling of spatially-resolved proteomics by direct TMT labeling tissue proteins on slides (Fig. 1). Isobaric labeling with TMT is used as a proof-of-principle study, TMT is a well-established robust system [45] for multiplex, relative protein quantitation of up to 18 samples. TMT binds to primary amines (N-terminal and epsilon amino group of lysine residues) in proteins/peptides using NHS chemistry [46]. Particularly, in protein labeling, TMT can be conjugated to accessible lysine residues and the protein N-terminus. SPOT utilizes TMT for the controlled labeling of proteins spatially distributed on a 2D-tissue slide. Subsequently, the entire tissue section would be harvested and subject to standard proteomic analysis workflow.

### TMT labeling at the protein-level on different tissue slides.

The efficacy of direct TMT labeling at the protein-level was evaluated across various slide types, including frozen tissues without staining, FFPE without staining, deparaffinized FFPE, deparaffinized and decrosslinked FFPE, and tissues with H&E staining, H staining, E staining. A mixture of 18 TMT tags was directly applied to the tissue sections, a pipette was used in this study as the initial applicator for tissue labeling. Subsequently, the entire tissue slide was scraped off, lysed, digested, and cleaned up using SCX followed by C18 STAGE tips and MS analysis. Upon completion of MS data generation, the raw data of each slide was searched and evaluated based on the identifications at PSM, peptide, and protein levels, as detailed in Table S4.

**Fig. 2:**
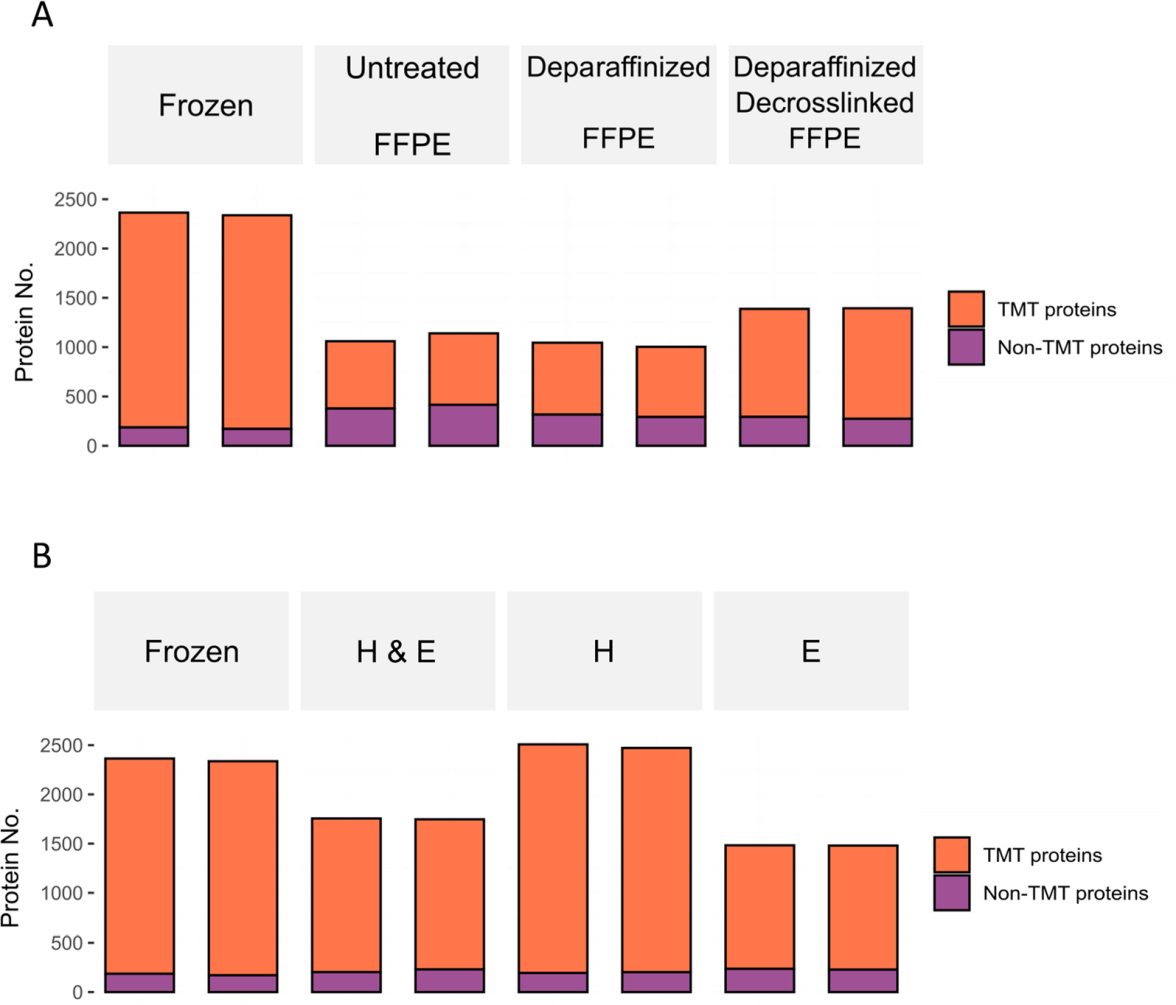
Total identified proteins from each type of sagittal mouse brain slide. **A** Protein identifications of frozen, untreated FFPE, deparaffinized FFPE, and deparaffinized/decrosslinked slides (all were unstained). **B** Protein identifications of frozen, H&E stained, H stained, and E stained slides

In general, tissue proteins on various types of slides could be labeled with TMT, with varying degrees of labeled protein percentages. Across all types of tissue slides, only 10% or less of the total PSMs were identified to have TMT tags at the protein N-termini. Consistency could be observed for the identifications at PSM, peptide, and protein levels between the two repeats of the same tissue slide type (Table S4). Frozen sagittal mouse brain slides served as a reference for assessing labeling efficiency, given that minimal treatment was applied to frozen slides. In comparison to frozen slides, where TMT labels were found on over 92% of proteins, a visible decrease in the percentage of labeled proteins could be observed in untreated, deparaffinized, and deparaffinized/decrosslinked slides (Fig. 2A). Remarkably, the labeling of proteins (~ 64%) is minimally affected by the presence of paraffin compared to the deparaffinized ones (~ 70%), supposedly attributable to the permeability of acetonitrile through the paraffin. Paraffin-coated surface also has low surface energy (high contact angle over 100°) [47], which limits the lateral spreading of the TMT solution. An adept control of TMT dot size is critical in ensuring precise and reproducible labeling efficiency in more precise settings such as labeling on tissue microarray (TMA) slides (coring size ~ 0.6 mm). In addition, the decrosslinking step also improved the labeling efficiency by ~ 8%.

Next, the effects of histology staining on direct tissue protein labeling were also evaluated (Fig. 2B). Notably, TMT tags were identified on over 92% of proteins from H-stained tissue slides. H&E stained (88%) and E-stained (84%) tissue slides showed a slightly lower percentage of labeled proteins compared to frozen and H-stained, however, the difference was less than 10%. During the H&E staining procedure, haemalum (oxidized hematoxylin solution) attaches to cell nuclei through covalent bonds between DNA phosphate oxygens and aluminum atoms, as well as between aluminum atoms and haemalum molecules. This covalent interaction between DNA and haemalum might release certain proteins bound to DNA, potentially elucidating the observed increase in protein identification with H-staining only. In contrast, eosin is attracted to tissue proteins by ionic forces (van der Waals forces) [48], and it could form salts with basic compounds like proteins. In turn, the presence of eosin could take up some of the binding capacity of SCX and C18 materials due to the hydrophobic interactions, providing a plausible explanation for the observed decrease in labeled protein percentage in H&E and E-stained slides.

### On-site labeling of proteins from different brain regions on mouse brain slide

**Fig. 3:**
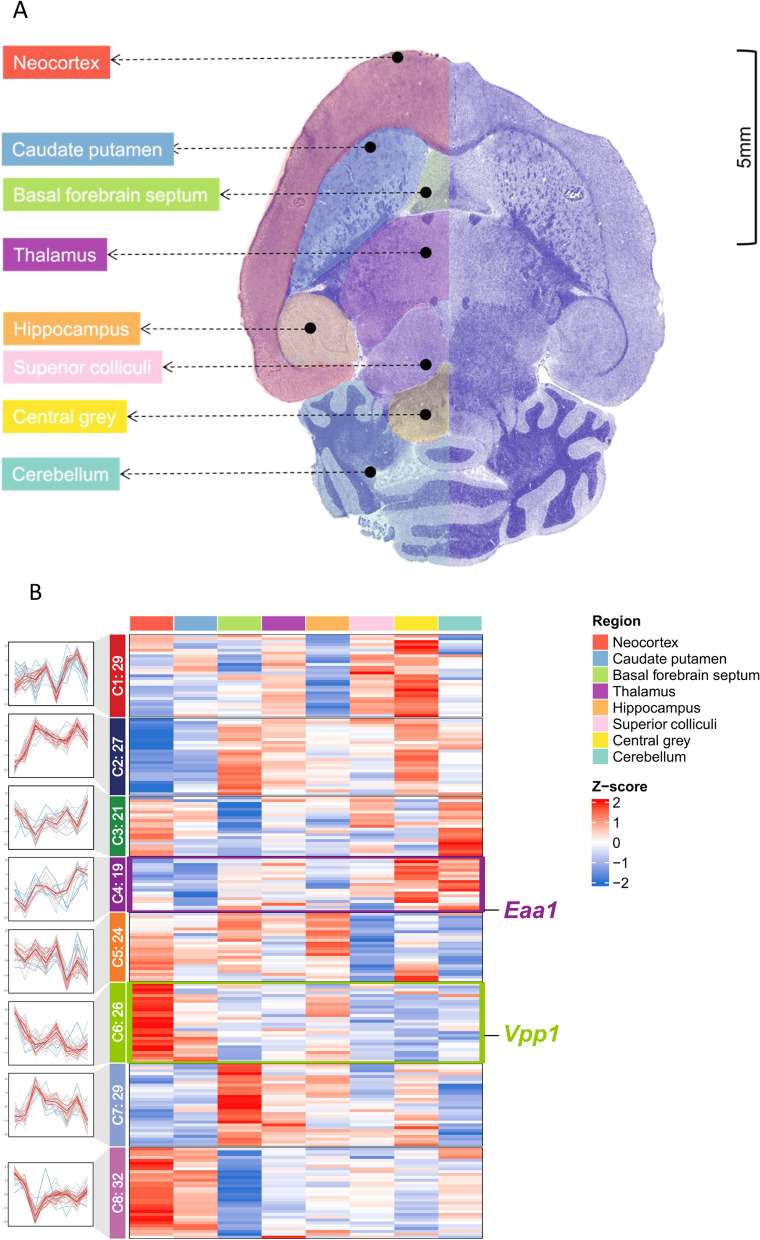
**A** Mouse brain slide in horizontal view. Eight different regions are color-coded as shown and a scale bar to show the size of the brain slide. The scanning image was augmented using the filter “Hematoxylin” and brain regions were marked using QuPath52 [49]. **B** Hierarchical clustering illustrating the proteomic quantification results across 8 brain regions. Protein expressions could be clustered into 8 clusters, each revealing a distinctive spatial trend displayed on the left side of the heatmap

Following the successful validation of direct labeling of tissue proteins on slides, we further evaluated SPOT's ability to detect proteomic patterns within a spatial context. A horizontal mouse brain slide with eight different regions was clearly outlined and each region was “stained” with a different TMT tag as illustrated in Fig. 3A. To enhance the visual recognition of the brain regions, the horizontal mouse brain slide was first stained with hematoxylin only, since hematoxylin did not interfere with the direct TMT labeling of tissue proteins (Fig. 2).

The mouse brain tissue was prepared similarly for downstream quantitative proteomics evaluation. Each region displayed a distinctive protein expression pattern and eight protein clusters were established using a soft clustering algorithm [50] (Fig. 3B, Figure S1, and Table S5). Among the eight clusters, cluster 4 (C4) and cluster 6 (C6) had obvious upward and downward protein expression trends starting from the neocortex to the cerebellum, respectively (Figs. 3B and S1). Excitatory amino acid transporter 1 (Eaa1), a glutamate transporter localized in the brain, was identified from C4 with the highest abundance in the cerebellum compared to the other regions, correlated well with a previous study showing that Eaa1 was highly enriched in the Purkinje cell layer in cerebellum [51]. On the other hand, elevated protein expression of V-type proton ATPase subunit a1 (Vpp1) in the neocortex was observed in C6. Vpp1 is reported to be predominantly expressed in neurons in the cortex and the dentate gyrus, part of the hippocampus. It can be found at low levels in astrocytes, oligodendrocytes, and microglia [52].

The results demonstrate that SPOT effectively detected proteomic patterns directly from tissue protein labeling indicating that SPOT is useful in studying spatial proteomics.

### On-site labeling of different Gleason score regions on the frozen slide and TMA slide

#### Prostate cancer frozen slide

**Fig. 4:**
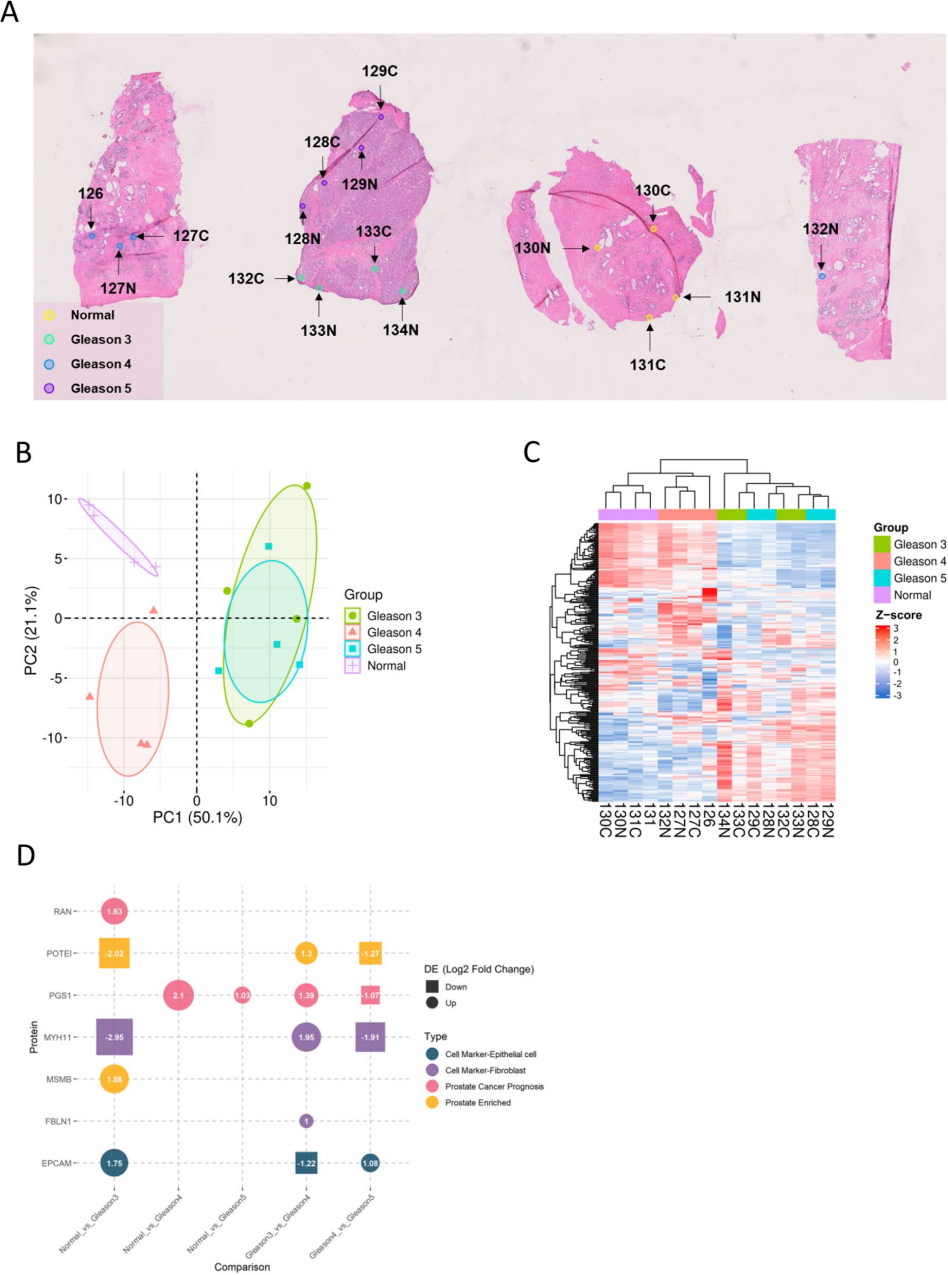
On-site TMT labeled frozen prostate cancer tissue slide. **A** Bright-field scanning of the adjacent prostate cancer H&E slide annotated with normal (yellow), Gleason 3 (cyan), Gleason 4 (blue), and Gleason 5 (purple) regions. **B** Principal component analysis of Gleason score regions based on the protein expression profiles. **C** Hierarchical clustering based on the expression profiles of 289 proteins across different Gleason score regions. **D** Significantly changed proteins (absolute log2 fold change > 1, p-value < 0.05) from pairwise comparison of two different Gleason score regions

To further test the on-site labeling on frozen tissue slides in discerning smaller regions of interest, an experienced pathologist annotated 4 regions of 0.6 mm in diameter within normal sections, Gleason score 3 sections, Gleason score 4 sections, and Gleason score 5 sections, respectively (Figs. 4A). Based on the pathological annotations on the adjacent H&E slides, direct TMT labeling was carried out on the frozen slides.

In total, 11,214 peptides were identified, corresponding to a set of 1,854 unique proteins. Within this dataset, 1,365 peptides were successfully labeled with TMT tags, corresponding to 289 distinct proteins. Following this identification, hierarchical clustering and principal component analysis (PCA) and hierarchical clustering were conducted to examine the association among different Gleason score regions based on their protein expression profiles (Fig. 4A, B). Notably, normal regions and Gleason4 regions could be separated completely, whereas Gleason 3 and Gleason 5 regions had a considerable overlap. Regions characterized by normal or the same Gleason score (ranging from Gleason 3 to 5) displayed notably high correlations across diverse tissue sections (Figure S2A). Conversely, regions associated with different Gleason scores exhibited relatively lower degrees of correlation.

Furthermore, differential analysis (Fig. 4D) was able to return two proteins specifically enriched in the prostate tissue (Human Proteome Atlas [2, 43]), two proteins that were found to relate to poor prognosis of prostate cancer (Human Proteome Atlas [2, 43]), and three proteins related to cell markers in the prostate (Cell Marker 2.0) [44]. Previous studies have indicated notable clinical relevance associated with microseminoprotein-beta (MSMB) [53] and epithelial cell adhesion molecule (EPCAM) [54, 55] for prostate cancer. In this study, MSMB was found to be overexpressed in Gleason 3 regions relative to normal regions, while EPCAM was found to be elevated in both Gleason 3 and Gleason 5 regions, but higher fold change was observed in Gleason 3 compared to normal (log2 fold change = 1.75) than Gleason 5 compared to Gleason 4 (log2 fold change = 1.08) (Fig. 4D). MSMB and EPCAM could be prostate cancer-relevant indicators or contributors in various medical and pathological conditions, underscoring the importance of further exploration and validation.

In summary, these results indicate that SPOT could capture potential correlations and variations in molecular profiles across different Gleason scores from frozen tissue slides even with direct TMT labeling of proteins in the 0.6 mm region.

### Prostate cancer TMA slide

**Fig. 5:**
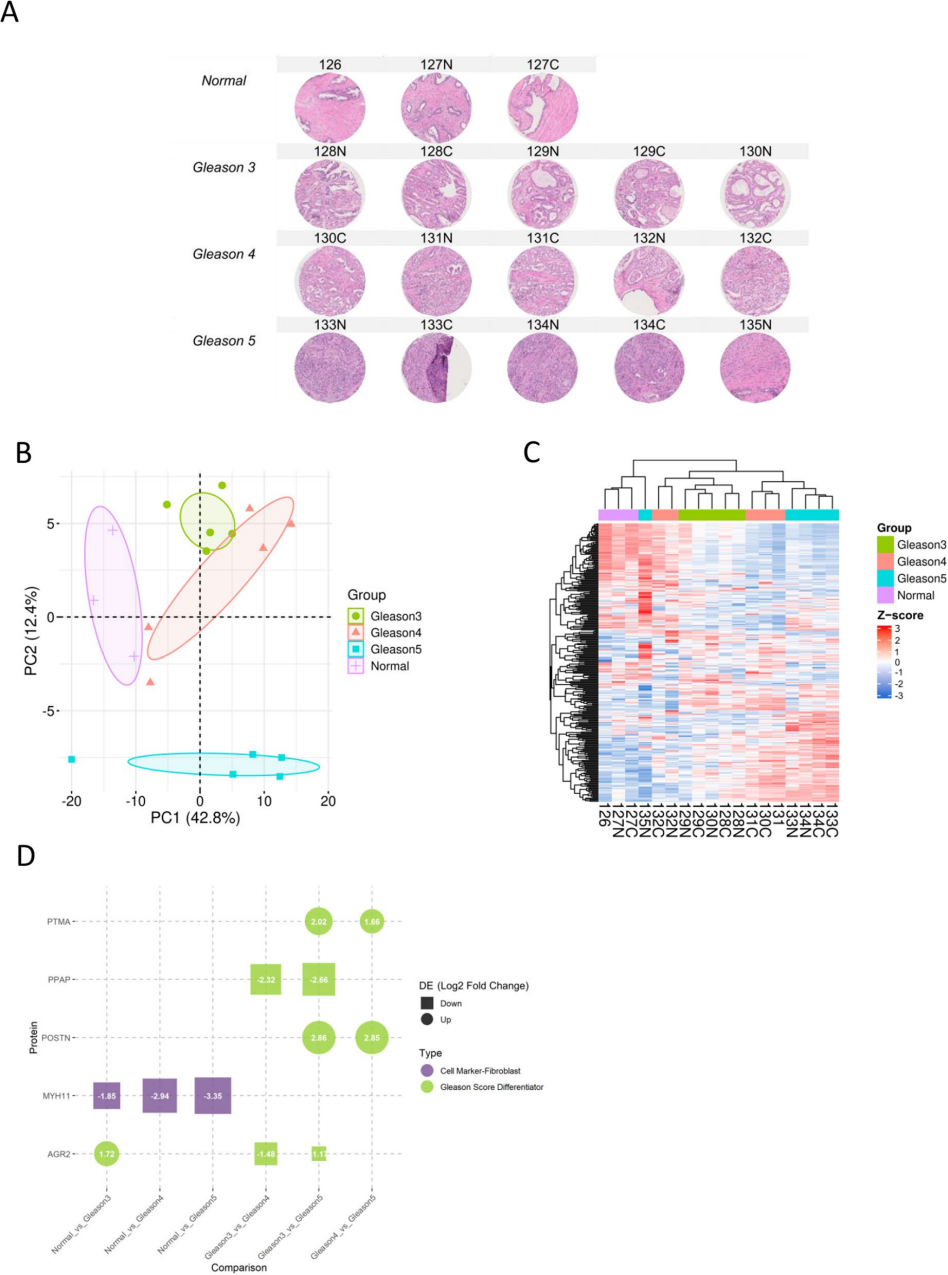
On-site TMT labeled prostate cancer TMA slide with paraffin. **A** Bright-field scanning image of selected cores from the adjacent H&E TMA of prostate cancer. Three normal cores, five Gleason score 3 cores, five Gleason score 4 cores and five Gleason score 5 cores were represented. **B** PCA analysis based on the protein expression profiles in different Gleason score regions. **C** Hierarchical clustering using the expression profiles of 265 proteins across different Gleason score regions. **D** Significantly changed proteins (absolute log2 fold change > 1, p-value < 0.05) from pairwise comparison of two different Gleason score cores

Following the application of TMT direct labeling onto frozen tissue slides derived from prostate cancer specimens, there arises a distinct interest in evaluating the translatability and consistency of this labeling methodology when extended to TMA slides. TMA cores were meticulously assessed by an experienced pathologist who assigned distinct scores to each core, based on the H&E stained adjacent slide (Fig. 5A). Eighteen cores of the size 0.6 mm were selected for TMT direct labeling (three for normal, five for Gleason score 3, five for Gleason score 4, and five for Gleason score 5).

The TMA format involves the systematic arrangement of discrete tissue cores, evenly spaced across the slide, providing a representative sampling of various specimens. Importantly, the deliberate spacing of these cores minimizes the risk of label mixing between different regions, ensuring a more accurate and region-specific evaluation of the TMT labeling method within the TMA framework. This comparative analysis aims to contribute valuable insights into the method's adaptability and reliability across different tissue slide formats, advancing our understanding of its applicability in broader histological contexts.

In total, 1,873 peptides (corresponding to 560 unique proteins) were identified, out of which 790 were TMT-labeled peptide sequences. These labeled peptides corresponded to 265 distinct proteins. Principal component analysis revealed minimal to no overlaps between each group (Fig. 5B), indicating distinct clustering patterns. The subsequent hierarchical clustering analysis (Fig. 5C) provided additional insight, revealing varying degrees of mixing between each group. This observation implies nuanced relationships and molecular heterogeneity within the regions characterized by different Gleason patterns.

Furthermore, the TMA dataset identified four proteins (PTMA, PPAP, POSTN, AGR2) that exhibited potential in distinguishing different Gleason score regions (Gleason score 3, 4, and 5). Additionally, one cell marker protein (MYH11) demonstrated the capability to differentiate normal regions from different Gleason score regions (Fig. 5C), a finding consistent with observations in the frozen dataset. These proteins identified in the TMA dataset, particularly the four proteins demonstrating variations among different Gleason score regions, suggest their potential for clinical applications in prostate cancer detection. Their implications in prostate cancer, as reported in previous studies [56–64], further underscore the significance of these proteins in the context of prostate cancer pathology. Understanding the molecular basis of Gleason patterns through these proteins could enhance the precision of prostate cancer grading. In addition, the cell marker protein capable of distinguishing normal from cancerous regions holds diagnostic potential and may serve as a valuable tool for clinicians in accurately identifying cancerous areas within the prostate.

Based on the correlation analysis result (Figure S2B), a pronounced association could be observed within normal cores as well as within Gleason score 3 cores. This strong correlation suggests a potential consistency in molecular characteristics within normal tissues and within low-grade prostate cancer. In contrast, the correlations observed among Gleason score 4 and Gleason score 5 cores exhibit greater variability, suggesting potential differences in tumor heterogeneity. The varied correlations observed in the higher-grade cores suggest the intricate nature of prostate tumor heterogeneity [56, 65]. This complexity arises from differences in the types and arrangements of cells, which can influence unique molecular characteristics within the tumor.

### Discussion and future directions

This study demonstrates a new approach, SPOT, for studying spatial proteomics quantitatively via direct labeling on tissue slides coupled with bottom-up MS. We were able to characterize different mouse brain regions as well as regions of different pathological states of prostate cancer directly from tissue slides of various forms by using SPOT. While these results are promising, further validation and optimization are necessary to fully exploit the potential of TMT labels for tissue slide analysis.

Applying labels directly onto tissue slides offers several advantages, including the ability to multiplex and analyze multiple samples in depth simultaneously, providing a comprehensive understanding of the tissue’s molecular landscape. Applying TMT directly onto proteins can present certain challenges. For instance, TMT can obstruct trypsin cleavage at lysine residues, resulting in longer peptides. To mitigate the effects from TMT protein-level labeling, utilization of alternative proteases may improve identification rate and coverage. Labeling at protein-level also grants us limited access to labeling sites, unlike peptide-level labeling, where TMT can easily target lysine residues and N-terminal sites. A peptide-level SPOT approach could overcome this limitation by applying a protease before TMT labeling. Additionally, SPOT was only applied to regions of interest, leaving most of the tissue slice unlabeled. We could improve the labeling efficiency by labeling the areas of interest with specific TMT channels in situ, then collecting the tissue and further labeling the samples with a different TMT tag in solution. Nonetheless, SPOT enables labeling the protein in its original context, enhancing the accuracy and relevance of the analysis.

As demonstrated using mouse brain and prostate cancer tissue slides, quantitatively profiling spatially distributed proteomes was achieved using SPOT. In the case of prostate cancer, the inherent heterogeneity of prostate tumors, characterized by diverse cellular populations and architectural patterns, poses a challenge for conventional Gleason scoring. While Gleason scoring remains a cornerstone in prostate cancer pathology, its limitations in fully capturing the intricacies of heterogeneous tumors are acknowledged. Ongoing research endeavors are dedicated to refining grading systems [66], particularly for tumors displaying mixed patterns, or using artificial intelligence-aided diagnosis [67, 68]. Our technology SPOT offers a viable solution for multiplexed spatial profiling using bottom-up proteomics and has the potential to unveil the complexities of prostate cancer heterogeneity. However, the current resolution of SPOT is insufficient to capture localized heterogeneity at the single-cell level. To address this limitation, exploring robust tissue labeling methods that offer enhanced precision and reproducibility is undoubtedly the next step. Techniques such as machine learning-assisted imaging processing and automated robotic arms equipped with high-precision imaging systems that can perform precise tissue manipulation and labeling may be considered. Future studies should also involve a larger sample size and include a broader range of pathological states to validate the observed differences and confirm the specificity and clinical utilities of the identified proteins.

Besides quantitatively profiling spatially distributed proteomes, we envision utilizing SPOT for the identification of protein–protein interactions and interactions with other binding partners (such as DNA, RNA, and metabolite) within specific subcellular compartments. Perturbations in the cellular microenvironment could induce alterations in the natural patterns of these interactions [69]. Such changes may arise as a consequence of environmental stressors or disease conditions, influencing the intricate network of interactions that govern cellular responses and functions. Identification of protein-binding partners at a spatial resolution is instrumental in deciphering not only the functionality of individual proteins but also the intricate protein pathways involved in biological and pathological processes.

In addition, the integration of spatial proteomics data with other omics data, such as genomics, transcriptomics, and metabolomics, can provide a comprehensive understanding of cellular processes and disease mechanisms. For example, the integration of spatial proteomics data with transcriptomics data can provide insight into the regulation of protein localization and expression, while the integration of spatial proteomics data with metabolomics data can provide insight into the functional consequences of alterations in protein localization or expression. As spatial transcriptomics advances towards unraveling the spatiotemporal intricacies of gene regulation [70], a similar perspective could be applied to spatial proteomics. In contrast to spatiotemporal transcriptomics, spatiotemporal proteomics has the potential to directly reveal the consequences of gene expression alterations across both spatial and temporal dimensions.

## Conclusions

SPOT reduces the complexity and time required for sample preparation, enabling high-throughput analysis of tissue samples more efficiently. SPOT provides a holistic view of the tissue proteome by capturing the proteomic profiles of entire tissue regions rather than isolated cell populations. This is advantageous for studying complex tissue interactions, and spatial relationships, and identifying global proteomic changes associated with pathological states, also advantageous in terms of sensitivity and dynamic range.

In conclusion, our study highlights the tremendous potential of utilizing SPOT for the in-depth investigation of the spatial distribution of the proteomes within biological and pathological contexts. Tissue slide analysis based on SPOT technology holds great promise for enhancing disease diagnosis, personalized medicine, and the development of targeted therapeutic strategies.

## Supplementary Information

Supplementary Material 1. Figure S1. Detailed clustering results of the protein expressions visualized using heatmap-GO-KEGG combination graph. Proteins exclusively or abundantly expressed in mouse brain are marked for each cluster. Spearman Correlation matrixes of the protein abundancesgenerated from the frozen prostate cancer tissue slides. A) Frozen slide. B) TMA slide

Supplementary Material 2

## Data Availability

Data is provided within the manuscript or supplementary information files.

## Abbreviations

SPOT: Spatial Proteomics through On-site Tissue Proteins Labeling
TMT: Tandem mass tag
H&E: Hematoxylin and eosin
H: Hematoxylin
E: Eosin
FFPE: Formalin-Fixed Paraffin-Embedded
TMA: Tissue microarray
DDA: Data-dependent acquisition
LMD: Laser microdissection
MS: Mass spectrometry
PSM: Peptide spectral match
IHC: Immunohistochemistry
IF: Immunofluorescence
MIBI: Multiplexed ion beam imaging
IMC: Imaging mass cytometry
