# Supplementary material for: SPOT: spatial proteomics through on-site tissue-protein-labeling
Source: Clin Proteomics. 2024 Oct 24;21:60. doi: 10.1186/s12014-024-09505-5 (PMC11515502; doi:10.1186/s12014-024-09505-5)
Supplement: Supplementary file 1 — Supplementary Material 1. Figure S1. Detailed clustering results of the protein expressions visualized using heatmap-GO-KEGG combination graph. Proteins exclusively or abundantly expressed in mouse brain are marked for each cluster. Spearman Correlation matrixes of the protein abundancesgenerated from the frozen prostate cancer tissue slides. A) Frozen slide. B) TMA slide [file 12014_2024_9505_MOESM1_ESM.pdf]

SPOT: Spatial Proteomics through On-site Tissue Proteins Labeling

Yuanwei Xu[1,2], T. Mamie Lih[1], Angelo M. De Marzo[1,3,4], Qing Kay Li[1,3*], Hui Zhang[1,2,3,4*]

Supplementary Graphs

Supplementary Graphs

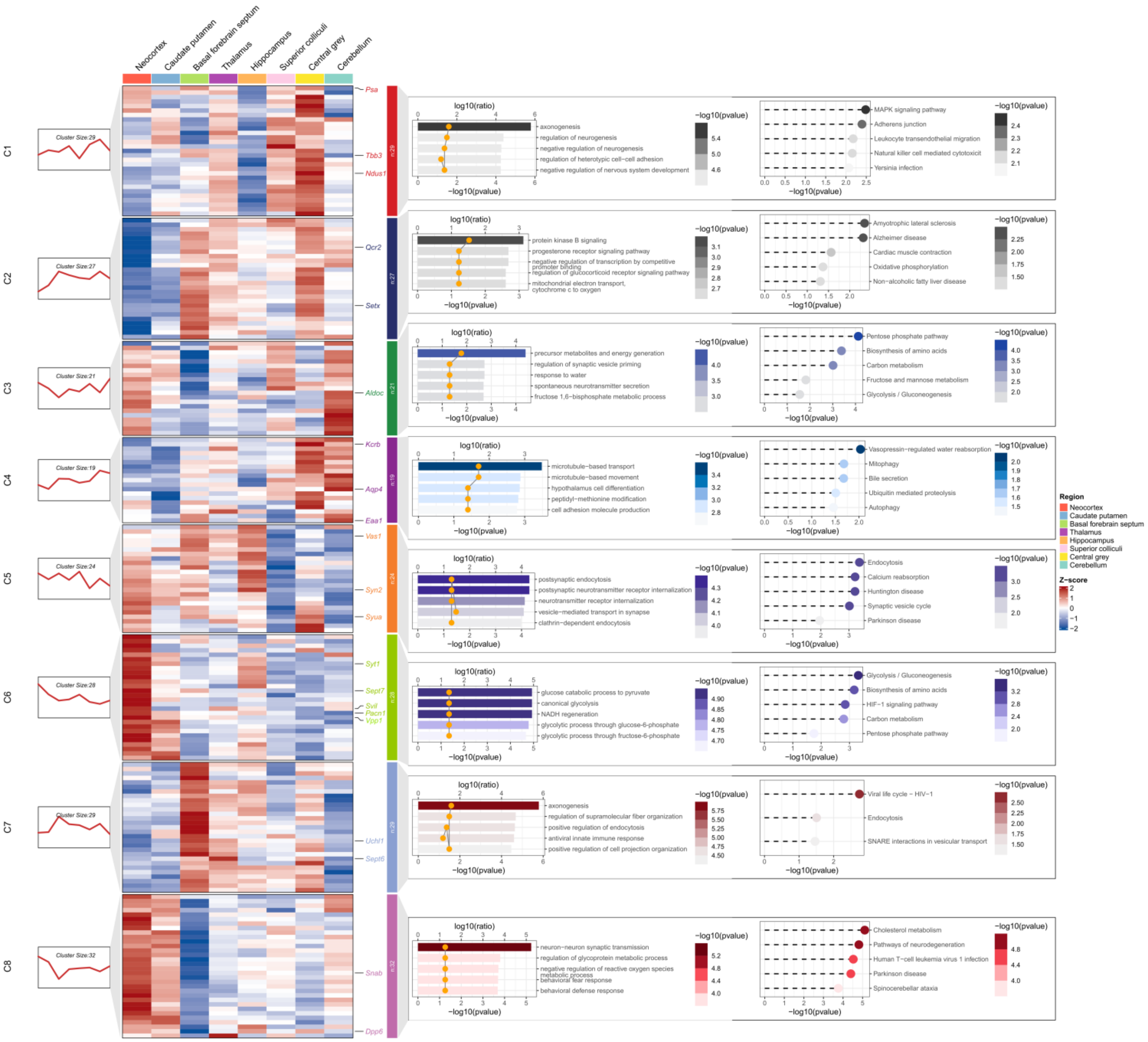

Figure S1. Detailed clustering results of the protein expressions visualized using heatmap-GO-KEGG combination graph. Proteins exclusively or abundantly expressed in mouse brain are marked for each cluster.

A

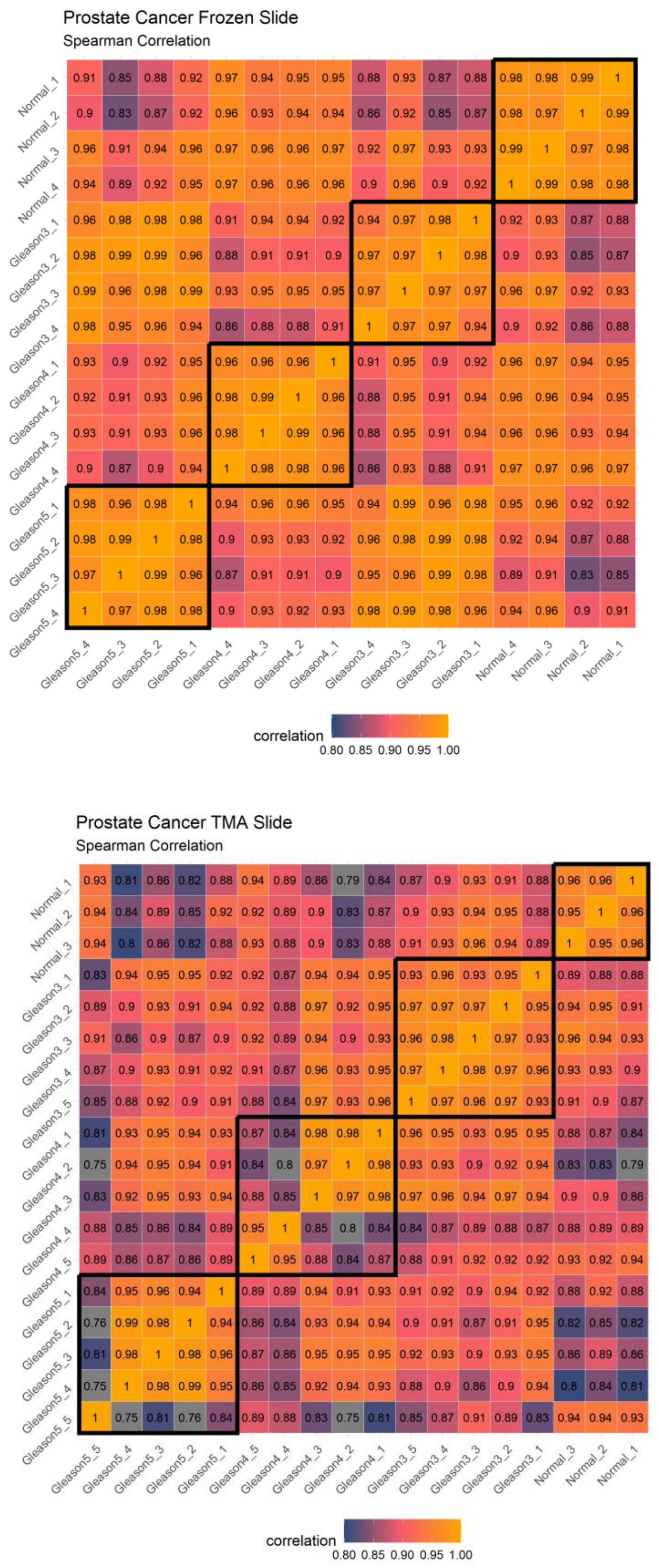

B

Figure S2. Spearman Correlation matrixes of the protein abundances (protein abundances were median-normalized) generated from the frozen prostate cancer tissue slides. A) Frozen slide. B) TMA slide.
